# Prevalence, trend, and associated risk factors for cleft lip with/without cleft palate: a national study on live births from 2016 to 2021

**DOI:** 10.1186/s12903-023-03797-z

**Published:** 2024-01-07

**Authors:** Mohammad-Hossein Heydari, Ali Sadeghian, Gita Khadivi, Hiba J. Mustafa, Ali Javinani, Nasser Nadjmi, Arash Khojasteh

**Affiliations:** 1 Department of Oral and Maxillofacial Surgery, Dental Research Center, Research Institute of Dental Sciences, Shahid Beheshti University of Medical Sciences, Chamran Highway, Velenjak St, Tehran, Iran; 2 Non-Communicable Diseases Research Center, Endocrinology and Metabolism Population Sciences Institute, Tehran University of Medical Sciences, Tehran, Iran; 3 Department of Obstetrics and Gynecology, Division of Maternal-Fetal Medicine, Indiana University School of Medicine, Indianapolis, IN USA; 4 Riley Children’s and Indiana University Health Fetal Center, Indianapolis, IN USA; 5 Maternal Fetal Care Center, Boston Children’s Hospital, Harvard Medical School, Boston, MA USA; 6 Department of Cranio-Maxillofacial Surgery/University Hospital, Faculty of Medicine & Health Sciences, University of Antwerp, Antwerp, Belgium; 7 Dental Research Center, Research Institute of Dental Sciences, Shahid Beheshti University of Medical Sciences, Tehran, Iran

**Keywords:** Cleft lip, Cleft palate, Congenital malformation, Congenital anomalies, Birth defects

## Abstract

**Backgrounds:**

Cleft lip with or without cleft palate (CL/P) is the most common congenital craniofacial anomaly, including non-syndromic cleft lip with or without cleft palate and cleft palate only. Failure in the fusion of median and lateral nasal processes, the maxillary prominence, and soft tissues around the oral cavity can cause CL/P. Previously, the prevalence has been estimated to be 1 among every 1000 births in 2014 among American neonates and no other reports have been available since. Thus, this study aimed to calculate the prevalence and trend of isolated CL/P among American live births from 2016 to 2021 with its associated risk factors.

**Methods and materials:**

In this cross-sectional population-based retrospective study, we used live birth data provided by the National Center for Health Statistics (NCHS) from the Center for Disease Control and Prevention (CDC). We calculated the prevalence per 10,000 live births of isolated (non-syndromic) CL/P from 2016 to 2021. To examine risk factors for developing isolated CL/P, we used logistic regression modelling.

**Results:**

The total prevalence per 10,000 births from 2016 to 2021 was 4.88 (4.79–4.97), for both sexes, and 5.96 (5.82–6.10) for males, and 3.75 (3.64–3.87) for females. The prevalence did not show any consistent linear decreasing or increasing pattern. We found significant association between increased odds of developing isolated CL/P among cases with 20 to 24 year-old mothers (OR = 1.07, 1.01–1.13, *p = 0.013*), mothers who smoked 11 to 20 cigarettes per day (OR = 1.46, 1.33–1.60, *p <  0.001*), mothers with extreme obesity (OR = 1.32, 1.21–1.43, *p <  0.001*), mothers with grade II obesity (OR = 1.32, 1.23–1.42, *p <  0.001*), mothers with pre-pregnancy hypertension (OR = 1.17, 1.04–1.31, *p = 0.009*), mothers with pre-pregnancy diabetes mellitus (OR = 1.96, 1.71–2.25, *p <  0.001*), and mothers who used assisted reproductive technology (OR = 1.40, 1.18–1.66, *p <  0.001*).

**Conclusions:**

Our findings suggest a minuscule increase, albeit insignificant, in the trend of CL/P prevalence from 2016 to 2021. Developing CL/P had greater odds among mothers with pre-pregnancy diabetes, smoking, obesity, and pre-pregnancy hypertension mothers along with mothers who used assisted reproductive technology. Isolated CL/P had the highest prevalence in non-Hispanic Whites, American Indian or Alaskan Native and Native Hawaiian and Other Pacific Islanders.

**Supplementary Information:**

The online version contains supplementary material available at 10.1186/s12903-023-03797-z.

## Introduction

Cleft lip with or without cleft palate (CL/P) is among the most common congenital malformations in oral and maxillofacial region which can be either isolated or associated with various syndromes. This condition can affect lips and maxilla along with soft and hard palate [1, 2]. In the sixth week of embryonic development, the upper lip takes form as the medial nasal processes fuse with the maxillary and lateral nasal processes. The fusion of the medial nasal processes in the midline shapes the inter-maxillary segment, creating the philtrum of the upper lip and the primary palate. The secondary palate begins to develop as bilateral projections from the maxillary processes. Initially, these palatal shelves grow vertically behind the primary palate and alongside the emerging tongue. During the eighth week of gestation, the palatal shelves reposition themselves above the tongue, starting from the anterior part of the palate and progressing towards the back. As development continues, the palatal shelves grow towards the midline and eventually fuse together [3–5]. Improper formation or fusion of the aforementioned structures, during these stages can lead to orofacial clefts. Although CL/P is not a fatal condition, however, CL/P-affected patients suffer from dental, occlusal, functional, and aesthetic problems along with secondary complications such as auditory, respiratory, and nutritional problems [6–8].

Environmental factors and genetics have been reported to have a significant association with CL/P [9]. Mutations in various genes have been previously observed among patients with CL/P, and is presented in numerous genetic syndromes [10]. More importantly, folic acid insufficiency has been suggested as a risk factor for oral clefts. Consumption of folic acid before and during early pregnancy reduces the chance of neural tube defects and oral clefts [11–15]. According to the latest study regarding the prevalence of CL/P among American mothers from 2010 to 2014, the total prevalence rate per 10,000 births reported to be 10.25 with the highest prevalence among non-Hispanic American Indians and non-Hispanic Alaska Natives (AIAN) [16]. However, no data was available in this study regarding the possible risk factors for CL/P [16].

To the best of our knowledge, no other study has reported the prevalence of CL/P among American mothers since then. Hence, this study aims at evaluating the prevalence and trend of isolated cases of CL/P affected pregnancies and its potential associated risk factors from 2016 to 2021, based on the annual birth data provided by the Center for Disease Control and Prevention (CDC).

## Methods and materials

### Data source and study design

This cross-sectional population-based study was designed using the birth data, also known as natality data, provided by the National Center for Health Statistics (NCHS) from the Center for Disease Control and Prevention (CDC). The standard certificate of birth is mandatory to be completed and publicly published for every birth occurring in the United States since 1968. The birth registration system collects data from 50 States, the independent registration of New York, and other districts. The birth data only includes births from US residents and non-residents inside the US. Births occurring to the US citizens or residents outside of the US is not included. This study used the anonymised, individual birth data from January 2016, to December 2021.

The NCHS provides separate certificates and reports for live birth, fetal death, or death. In this study, we used the live birth data. Live birth was defined as a new-born with any sign of life after delivery, regardless of the length of pregnancy [17]. The live birth data is collected based on this definition and is precisely distinctive from fetal death. Further information, regarding the birth certificate, data collection, and modelling procedures are available elsewhere [18].

### Exposure variables

The following variables were extracted and cleaned from the CDC dataset: [1] demographic variables including birth year, maternal age, race/ethnicity, education, payment source for delivery, sex of the infant, [2] perinatal variables including pre-pregnancy body mass index (BMI), pre-pregnancy smoking, infertility treatment use (fertility enhancing drugs, assistive reproductive technology, or both), previous pre-term delivery, pre-pregnancy diabetes mellitus, pre-pregnancy hypertension, and [3] congenital anomalies such as anencephaly, meningomyelocele/spina bifida, cyanotic congenital heart disease, congenital diaphragmatic hernia, omphalocele, gastroschisis, limb reduction defect, cleft lip or palate, Down syndrome, and suspected chromosomal disorder.

Isolated CL/P was defined as a living birth with CL/P and without any other aforementioned congenital anomalies. Non-isolated CL/P cases were excluded from our study. BMI was categorised as underweight (< 18.5), normal (18.5–24.9), overweight (25–29.9), obese I (30–34.9), obese II (35–39.9), and extremely obese (≥ 40). Age was also categorised as under 20 years old, 20 to 24 years old, 25 to 29 years old, 30 to 34 years old, 35 to 39 years old, and over 40 years old. Pre-pregnancy smoking (number of cigarettes per day) was categorised into no (zero cigarettes), 1–5 cigarettes per day, 6–10 cigarettes per day, 11–20 cigarettes per day, and > 20 cigarettes per day [19]. The CDC data provides different classifications for maternal race/ethnicity. We used the data for maternal race and Hispanic origin according to previous studies [16, 20], based on which all cases were categorized into non-Hispanic (NH) white, NH Black, NH Asian, Hispanic, and NH others. The latter includes non-Hispanic American Indian or Alaskan Native (AIAN) and non-Hispanic Native Hawaiian and Other Pacific Islanders (NHOPI), which were combined due to lower number of cases compared to the other races/ethnicities.

### Statistical analysis

We calculated the total and annual prevalence rate per 10,000 births for isolated CL/P from 2016 to 2021 based on the aforementioned independent variables. To detect any significant increasing or decreasing trend in each category, a Cochran-Armitage test of trend was performed. The Cochran-Armitage test of trend is used to detect any increasing or decreasing trend of the probability of positive outcomes for binary variables (like mortality, having CL/P, etc.) in ordered groups (in our case, the consecutive years from 2016 to 2021). In simpler words, it tests whether a certain distribution of the positive outcomes (CL/P) can be found based on the ordered group variable (year). However, we can limit the groups, in which the prevalence is being compared throughout time. Hence, it is possible to compare the prevalence among each specific group, from 2016 to 2021 (Table 1). We also used logistic regression modelling to evaluate the association of certain potential risk factors (maternal age, race/ethnicity, smoking, BMI, pre-pregnancy diabetes, pre-pregnancy hypertension, previous preterm birth, and infertility treatment use) and the occurrence of CL/P. Initially, we added the independent variables into a univariate logistic model to provide the crude odds ratios and *p*-values. In the next step, those independent variables with *p*-values less than 0.01 were added into the adjusted multivariable logistic model. STATA version 17 (StataCorp LLC), R (R Foundation for Statistical Computing, Vienna, Austria), and RStudio (RStudio, Inc., Boston, MA) were used for data cleaning, data analysis, and creating the Figs. *P*-values less than 0.05 were considered as statistically significant.

**Table 1:** Prevalence of isolated CLP affected pregnancies per 10,000 births (95% CI)

		Total	2016	2017	2018	2019	2020	2021	p-value^*^
**Total**		4.88 (4.79–4.97)	5.02 (4.80–5.24)	4.85 (4.64–5.08)	5.11 (4.88–5.34)	4.63 (4.41–4.85)	4.71 (4.49–4.94)	4.94 (4.72–5.17)	***0.027***
**Maternal Age**	*Less than 20*	5.29 (4.87–5.75)	5.23 (4.3–6.3)	4.93 (4–6.02)	5.23 (4.23–6.39)	5.64 (4.58–6.87)	4.94 (3.91–6.15)	5.91 (4.74 7.28)	*0.428*
	*20 to 24*	5.48 (5.26–5.71)	6 (5.48–6.56)	5.37 (4.86–5.91)	5.67 (5.13–6.24)	5.19 (4.67–5.75)	5.12 (4.59–5.69)	5.46 (4.91–6.06)	*0.074*
	*25 to 29*	5 (4.83–5.18)	4.93 (4.53–5.35)	5.2 (4.85–5.72)	5.27 (4.85–5.72)	4.78 (4.37–5.21)	4.58 (4.18–5.02)	5.24 (4.81–5.02)	*0.080*
	*30 to 34*	4.46 (4.3–4.62)	4.54 (4.16–4.96)	4.3 (3.92–4.71)	4.67 (4.27–5.09)	4.37 (3.98–4.78)	4.56 (4.17–4.99)	4.3 (3.92–4.7)	*0.654*
	*35 to 39*	4.5 (4.298–4.73)	4.34 (3.8–4.93)	4.5 (3.96–5.09)	4.84 (4.29–5.45)	3.94 (3.44–4.49)	4.57 (4.03–5.16)	4.79 (4.25–5.39)	*0.492*
	*Over 40*	5.18 (4.68–5.71)	6.37 (5.03–7.95)	4.9 (3.75–6.29)	5.34 (4.15–6.77)	4.3 (3.25–5.59)	5.23 (4.06–6.63)	5.01 (3.89–6.35)	*0.208*
	***p-value***^***҂***^	***< 0.001***	***0.001***	***0.006***	***0.028***	***< 0.001***	*0.342*	***0.003***	*–*
**Maternal Race/Ethnicity**	*NH*^*a*^ *White*	5.51 (5.38–5.65)	5.71 (5.39–6.05)	5.48 (5.16–5.82)	5.73 (5.4–6.08)	5.37 (5.04–5.7)	5.28 (4.96–5.63)	5.48 (5.15–5.82)	*0.132*
	*NH Black*	2.89 (2.71–3.08)	2.93 (2.5–3.42)	2.8 (2.38–3.27)	2.95 (2.52–3.44)	2.86 (2.43–3.35)	2.45 (2.05–2.91)	3.34 (2.86–3.82)	*0.122*
	*NH Asian*	3.84 (3.52–4.17)	3.81 (3.09–4.64)	4.36 (3.58–5.27)	3.73 (3–4.59)	3.09 (2.43–3.88)	4.38 (3.54–5.34)	3.65 (2.88–4.55)	*0.135*
	*Hispanic*	4.82 (4.64–5.01)	4.89 (4.45–5.36)	4.8 (4.36–5.27)	5.23 (4.76–5.72)	4.39 (3.96–4.87)	4.81 (4.36–5.28)	4.80 (4.36–5.28)	*0.181*
	*NH Others*	6.34 (5.78–6.94)	6.41 (5.07–8)	5.42 (4.19–6.89)	7.02 (5.62–8.67)	6.53 (5.17–8.12)	5.72 (4.45–7.23)	6.93 (5.54–8.57)	*0.436*
**Maternal Education**	*< 12 years*	6.09 (5.8–6.38)	6.28 (5.63–6.99)	5.49 (4.86–6.17)	6.32 (5.62–7.07)	5.81 (5.13–6.56)	5.74 (5.04–6.51)	6.98 (6.19–7.85)	*0.286*
	*> 12 years*	4.71 (4.61–4.81)	4.82 (4.59–5.06)	4.75 (4.51–4.99)	4.94 (4.7–5.19)	4.47 (4.24–4.7)	4.58 (4.34–4.82)	4.69 (4.46–4.93)	*0.099*
**Smoking before Pregnancy**^***⁑***^	*No*	4.62 (4.53–4.71)	4.74 (4.52–4.97)	4.58 (4.36–4.81)	4.81 (4.58–5.04)	4.48 (4.26–4.71)	4.41 (4.19–4.64)	4.69 (4.46–4.92)	*0.114*
	*1–5*	6.82 (6.1–7.61)	7.05 (5.48–8.94)	6.76 (5.17–8.69)	7.77 (5.99–9.9)	5.36 (3.85–7.27)	6.62 (4.84–8.83)	7.34 (5.31–9.89)	*0.440*
	*6–10*	7.76 (7.08–8.5)	6.96 (5.58–8.58)	7.41 (5.92–9.15)	8.24 (6.61–10.15)	7.12 (5.54–9.01)	8.55 (6.7–10.75)	8.99 (6.92–11.48)	*0.109*
	*11–20*	8.53 (7.81–9.3)	8.22 (6.71–9.96)	7.91 (6.39–9.7)	8.91 (7.22–10.88)	6.66 (5.13–8.51)	10.14 (8.1–12.53)	10.14 (7.94–12.77)	*0.121*
	*> 20*	7.76 (6.47–9.23)	8.6 (5.99–11.96)	7.85 (5.34–11.15)	7.42 (4.89–10.79)	5.74 (2.75–10.56)	7.53 (3.89–13.15)	8.73 (4.51–15.24)	*0.672*
	***p-value***^***҂***^	***< 0.001***	***< 0.001***	***< 0.001***	***< 0.001***	***< 0.001***	***< 0.001***	***< 0.001***	*–*
**BMI**	*Underweight*	4.87 (4.36–5.42)	4.75 (3.65–6.06)	4.4 (3.32–5.72)	5.16 (3.95–6.63)	4.83 (3.63–6.31)	5.4 (4.06–7.05)	4.78 (3.5–6.38)	*0.584*
	*Normal*	4.45 (4.32–4.59)	4.59 (4.27–4.92)	4.71 (4.38–5.06)	4.47 (4.15–4.82)	4.37 (4.04–4.71)	4.2 (3.87–4.55)	4.32 (3.99–4.68)	***0.038***
	*Overweight*	4.46 (4.49–4.84)	4.89 (4.47–5.35)	4.47 (4.06–4.91)	4.76 (4.34–5.21)	4.23 (3.83–4.66)	4.63 (4.21–5.09)	4.99 (4.56–5.45)	*0.805*
	*Obesity I*	5.27 (5.03–5.52)	5.63 (5.02–6.29)	4.8 (4.24–5.42)	5.59 (5.37–6.67)	5.21 (4.63–5.84)	5.02 (4.45–5.64)	5.01 (4.46–5.62)	*0.204*
	*Obesity II*	5.99 (5.63–6.37)	6.46 (5.53–7.5)	6.3 (5.39–7.32)	6.57 (5.65–7.6)	4.91 (4.13–5.79)	5.4 (4.59–6.31)	6.35 (5.47–7.32)	*0.224*
	*M-Obese*^*b*^	6.1 (5.67–6.55)	6.02 (4.96–7.23)	6.26 (5.2–7.48)	6.67 (5.59–7.9)	5.88 (4.88–7.03)	6.04 (5.03–7.19)	5.75 (4.79–6.86)	*0.526*
	***p-value***^***҂***^	***< 0.001***	***< 0.001***	***< 0.001***	***< 0.001***	**0.001**	***< 0.001***	***< 0.001***	*–*
**Pre– pregnancy Diabetes**	*Yes*	10.82 (9.48–12.28)	9.18 (6.23–12.02)	8.78 (5.96–12.46)	14.28 (10.63–18.77)	10.12 (7.13–13.95)	12.61 (9.3–16.72)	9.83 (6.99–13.43)	*0.503*
	*No*	4.82 (4.73–4.91)	4.97 (4.75–5.2)	4.82 (4.6–5.05)	5.01 (4.79–5.25)	4.58 (4.36–4.8)	4.63 (4.41–4.86)	4.88 (4.65–5.11)	*0.111*
**Pre– pregnancy Hypertension**	*Yes*	6.38 (5.69–7.12)	5.85 (4.18–7.97)	6.44 (4.73–8.56)	7.28 (5.51–9.43)	6.74 (5.09–8.75)	6.67 (5.2–8.56)	5.42 (4.07–7.08)	*0.655*
	*No*	4.84 (4.75–4.93)	4.99 (4.77–5.22)	4.83 (4.61–5.05)	5.06 (4.83–5.29)	4.58 (4.37–4.81)	4.66 (4.44–4.89)	4.92 (4.69–5.15)	*0.151*
**Infertility Treatment Use**	*No Treatment*	4.86 (4.77–4.96)	4.99 (4.77–5.22)	4.84 (4.62–5.07)	5.09 (4.86–5.32)	4.63 (4.42–4.86)	4.7 (4.47–4.93)	4.91 (4.69–5.15)	***0.048***
	*Fertility Enhancing Drugs*	4.52 (3.48–5.78)	4.12 (1.97–7.58)	6.48 (3.7–10.52)	4.9 (2.53–8.56)	3.45 (1.49–6.8)	4.03 (1.84–7.65)	3.99 (1.82–7.58)	*0.413*
	*Asst. Reproductive Technology*	6.18 (5.23–7.25)	7.92 (5.22–11.53)	5.41 (3.3–8.35)	6.67 (4.36–9.77)	5.1 (3.2–7.73)	5.89 (3.81–8.69)	6.38 (4.33–9.05)	*0.523*
	*Both*	6.59 (4.18–9.89)	5.52 (1.14–1.16)	3.41 (4.14–12.34)	5.09 (1.05–14.88)	5.13 (1.05–14.98)	12.91 (5.19–26.59)	7.74 (2.51–18.06)	*0.171*
**Payment Source for Delivery**	*Medicaid*	5.46 (5.31–5.61)	5.71 (5.36–6.09)	5.35 (5.01–5.72)	5.84 (5.47–6.23)	5.12 (4.77–5.49)	5.17 (4.81–5.55)	5.53 (− 5.16–5.92)	*0.149*
	*Private Insurance*	4.42 (4.29–4.54)	4.48 (4.19–4.79)	4.48 (4.18–4.79)	4.51 (4.21–4.82)	4.23 (3.94–4.53)	4.28 (3.99–4.6)	4.51 (4.21–4.83)	*0.585*
	*Self– pay*	4.85 (4.42–5.31)	4.75 (3.76–5.91)	4.41 (3.45–5.56)	4.49 (3.52–5.65)	4.49 (3.52–5.65)	5.3 (4.18–6.63)	4.97 (3.89–6.26)	*0.523*
	*Others*	4.85 (4.38–5.34)	4.36 (3.38–5.53)	4.88 (3.82–6.15)	5.12 (4.03–6.42)	4.94 (3.81–6.3)	5.22 (4.02–6.65)	4.62 (3.51–5.97)	*0.632*
**Previous Preterm Birth**	*Yes*	7.04 (6.46–7.64)	6.47 (5.12–8.07)	6.96 (5.58–8.57)	8.21 (6.75–9.89)	6.15 (4.91–7.62)	6.21 (4.95–7.7)	8.12 (6.7–9.77)	*0.513*
	*No*	4.80 (4.71–4.89)	4.96 (4.74–5.19)	4.79 (4.57–5.01)	4.99 (4.76–5.22)	4.57 (4.35–4.8)	4.66 (4.43–4.89)	4.80 (4.58–5.04)	*0.093*
**Sex of Infant**	*Male*	5.96 (5.82–6.1)	6.92 (5.95–6.65)	5.81 (5.48–6.16)	6.15 (5.81–6.51)	5.6 (5.27–5.95)	5.79 (5.45–6.15)	6.08 (5.74–6.45)	*0.239*
	*Female*	3.75 (3.64–3.87)	3.68 (3.41–3.96)	3.85 (3.57–4.14)	4.02 (3.74–4.32)	3.61 (3.34–3.9)	3.59 (3.31–3.88)	3.74 (3.47–4.04)	*0.479*

^a^ Non–Hispanic, ^b^ morbidly obese, *p-value for Cochrane Armitage test of trend across the time from 2016 to 2021, **⁑** number of cigarettes per day, ҂ Cochran-Armitage test of trend across different categories in each year

## Results

Out of 22,651,555 live births from January 2016 to December 2021, 17,872 records were excluded due to missing data for CL/P. Also, 11,054 and 653 records had isolated and non-isolated CL/P, respectively. The non-isolated records were excluded from our study. Overall, 418 records had missing data for the other independent variables which were also removed. Finally, 10,636 records with isolated CL/P were included in the analysis.

**Fig. 1:**
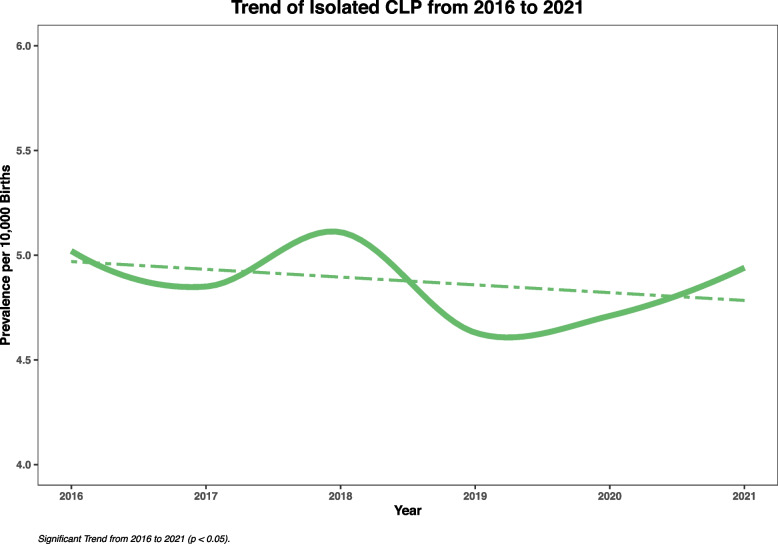
Trend of Isolated CL/P from 2016 to 2021

The total prevalence of isolated CL/P was 4.88 per 10,000 births (95% CI: 4.79–4.97) from 2016 to 2021. The prevalence was 5.02 per 10,000 births (95% CI: 4.80–5.24) in 2016 compared to 4.94 per 10,000 births (95% CI: 4.72–5.17) in 2021. The prevalence was 5.96 per 10,000 births (95% CI: 5.82–6.10) for males and 3.75 per 10,000 births (95% CI: 3.64–3.87) for females. Albeit not significantly, the prevalence decreased among males from 2016 to 2021, however, it was fairly stable among females The prevalence underwent both decrease and increase from 2016 to 2021 and did not show any significant linear decreasing or increasing pattern, however, based on the test of trend, there was a significant non-linear pattern from 2016 to 2021 (Fig. 1). More detail regarding the prevalence of isolated CL/P from 2016 to 2021 is available in Table 1. Also, further detail regarding the prevalence of isolated CL/P in different maternal age and race groups is summarised in Appendix 1 and 2.

**Fig. 2:**
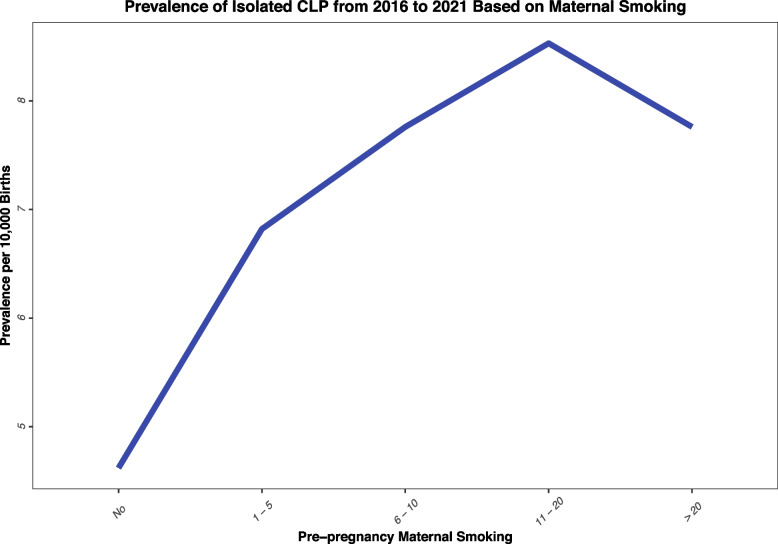
Prevalence of Isolated CL/P from 2016 to 2021 Based on Maternal Smoking

**Fig. 3:**
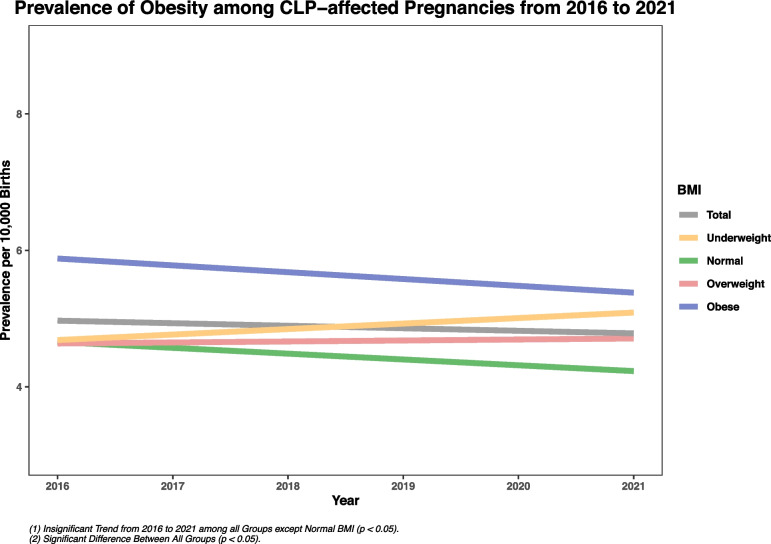
Prevalence of Obesity among CL/P − affected Pregnancies from 2016 to 2021

**Fig. 4:**
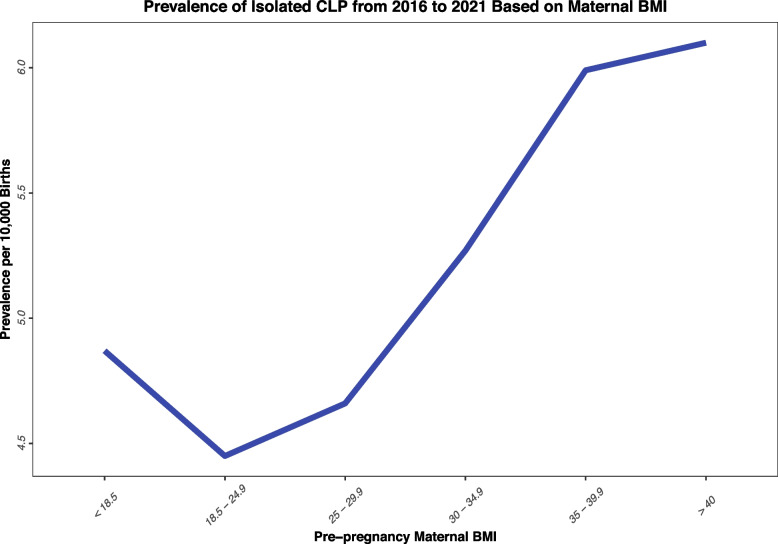
Prevalence of Isolated CL/P from 2016 to 2021 Based on Maternal BMI

**Fig. 5:**
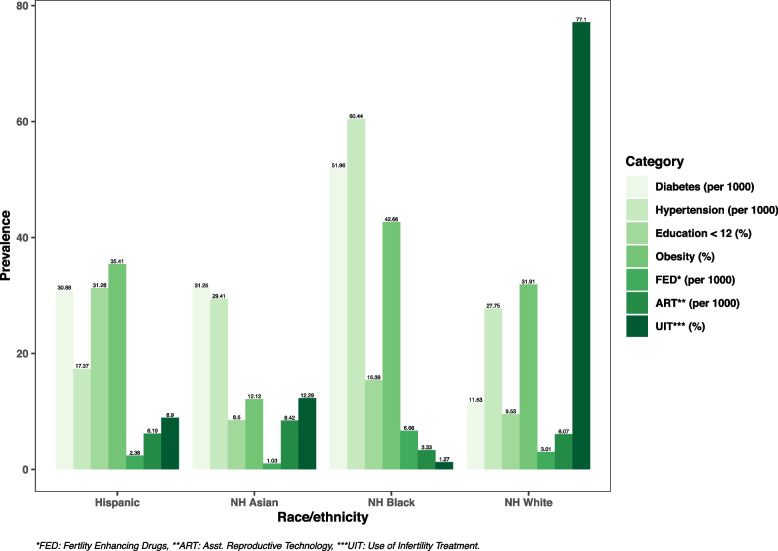
Comparing the Prevalence of Isolated CL/P among Different Races/Ethnicities Based on Certain Characteristics

The prevalence of isolated CL/P was the highest among mothers with 11 to 20 cigarettes smoking per day compared to non-smoker mothers. It should be noted that the prevalence rate for all smoking groups increased from 2016 to 2021, while the prevalence decreased for the non-smoking mothers (Table 1, Appendix 3). Figure 2 presents the prevalence between different frequencies of pre-pregnancy smoking. Among different BMI groups, the highest prevalence was among mothers with extreme obesity (6.10, 95% CI: 5.67–6.55), followed by mothers with grade II obesity (5.99, 95% CI: 5.63–6.37), and mothers with grade I obesity (5.27, 95% CI: 5.03–5.52) (Figs. 3, 4, 5, Appendix 4).

Regarding the risk factors for isolated CL/P in the multivariable adjusted model, mothers who were 20 to 24 years old had a significantly higher risk for having a child with isolated CL/P (OR = 1.07, 95% CI: 1.01–1.13, *p-value = 0.013*), compared to mothers who were 25 to 29 years old. Also, mothers who were 30 to 34 (OR = 0.91, 95% CI: 0.87–0.96, *p-value = 0.001*), and 35 to 39 (OR = 0.91, 95% CI: 0.85–0.97, *p-value = 0.005*) had significantly lower risk for having a child isolated CL/P. Among different races/ethnicities, NH Black mothers (OR = 0.47, 95% CI: 0.44–0.50, *p-value <  0.001*), NH Asian mothers (OR = 0.79, 95% CI: 0.72–0.86, *p-value <  0.001*), and Hispanic mothers (OR = 0.80, 95% CI: 0.76–0.84, *p-value <  0.001*) had lower risk for having a child with isolated CL/P compared to NH White mothers. Although NH NHOPI and AIAN mothers had a significantly higher risk for having a child with isolated CL/P in the univariate model (OR = 1.14, 95% CI: 1.04–1.26, *p-value <  0.004*), however, this effect faded in the adjusted multivariable model adjustment (OR = 1.02, 95% CI: 0.92–1.12, *p-value = 0.662*).

Based on the results of the multivariable model, smoking and obesity were both associated with higher risk of developing isolated CL/P. Mothers who smoked 11 to 20 cigarettes per day had the highest risk (OR = 1.46, 95% CI: 1.33–1.60, *p-value <  0.001*) for having a child with isolated CL/P. Also, mothers with extreme obesity (OR = 1.32, 95% CI: 1.21–1.43, *p-value <  0.001*) and mothers with grade II obesity (OR = 1.32, 95% CI: 1.23–1.42, *p-value <  0.001*) had also higher risk for developing isolated CL/P. Mothers with pre-pregnancy hypertension (OR = 1.17, 95% CI: 1.04–1.31, *p-value = 0.009*), mothers with pre-pregnancy diabetes (OR = 1.96, 95% CI: 1.71–2.25, *p-value <  0.001*), and mothers with previous pre-term birth (OR = 1.41, 95% CI: 1.29–1.54, *p-value <  0.001*) had all higher risk for having a child with isolated CL/P before and after adjustment. It should also be noted that among mothers who received infertility treatment, only those who received assisted reproductive technology treatment had a significantly higher chance of having a child with isolated CL/P (OR = 1.40, 95% CI: 1.18–1.66, *p-value <  0.001*). Further details, regarding the univariate and the multivariate models are available in Table 2.

**Table 2:** Univariable and multivariable logistic regression model of risk factors associated with isolated CLP

		Crude OR	*p-value*	Adjusted^a^ OR	*p-value*
**Maternal Age**	*Less than 20*	1.05 (0.96–1.15)	*0.216*	1.02 (0.93–1.13)	*0.547*
	*20 to 24*	1.09 (1.03–1.15)	***0.001***	1.07 (1.01–1.13)	***0.013***
	*25 to 29*	1.0 (ref^b^)	*–*	1.0 (ref)	*–*
	*30 to 34*	0.89 (0.84–0.93)	***< 0.001***	0.91 (0.87–0.96)	***0.001***
	*35 to 39*	0.89 (0.84–0.95)	***0.001***	0.91 (0.85–0.97)	***0.005***
	*Over 40*	1.03 (0.93–1.14)	*0.512*	1.04 (0.93–1.16)	*0.423*
**Maternal Race/Ethnicity**	*NH White*^*c*^	1.0 (ref)	*–*	1.0 (ref)	*–*
	*NH Black*	0.52 (0.48–0.56)	***< 0.001***	0.47 (0.44–0.50)	***< 0.001***
	*NH Asian*	0.69 (0.63–0.75)	***< 0.001***	0.79 (0.72–0.86)	***< 0.001***
	*Hispanic*	0.87 (0.83–0.91)	***< 0.001***	0.80 (0.76–0.84)	***< 0.001***
	*Others*	1.14 (1.04–1.26)	***0.004***	1.02 (0.92–1.12)	*0.662*
**Smoking before Pregnancy** (Number of cigarettes per day)	*No*	1.0 (ref)	*–*	1.0 (ref)	*–*
	*1–5*	1.47 (1.32–1.64)	***< 0.001***	1.26 (1.12–1.41)	***< 0.001***
	*6–10*	1.68 (1.53–1.84)	***< 0.001***	1.37 (1.24–1.51)	***< 0.001***
	*11–20*	1.84 (1.68–2.01)	***< 0.001***	1.46 (1.33–1.60)	***< 0.001***
	*> 20*	1.67 (1.40–2.00)	***< 0.001***	1.42 (1.18–1.71)	***< 0.001***
**BMI**	*Underweight*	1.09 (0.97–1.22)	*0.118*	1.01 (0.90–1.13)	*0.802*
	*Normal*	1.0 (ref)	*–*	1.0 (ref)	*–*
	*Overweight*	1.04 (0.99–1.09)	*0.062*	1.06 (1.00–1.11)	***0.019***
	*Obesity I*	1.18 (1.12–1.25)	***< 0.001***	1.18 (1.12–1.25)	***< 0.001***
	*Obesity II*	1.34 (1.25–1.44)	***< 0.001***	1.32 (1.23–1.42)	***< 0.001***
	*Ex Obese* ^*d*^	1.36 (1.26–1.48)	***< 0.001***	1.32 (1.21–1.43)	***< 0.001***
**Pre-pregnancy Diabetes**	*No*	1.0 (ref)	*–*	1.0 (ref)	*–*
	*Yes*	2.24 (1.97–2.55)	***< 0.001***	1.96 (1.71–2.25)	***< 0.001***
**Pre-pregnancy Hypertension**	*No*	1.0 (ref)	*–*	1.0 (ref)	*–*
	*Yes*	1.31 (1.17–1.47)	***< 0.001***	1.17 (1.04–1.31)	***0.009***
**Previous Preterm Birth**	*No*	1.0 (ref)	*–*	1.0 (ref)	*–*
	*Yes*	1.46 (1.34–1.59)	***< 0.001***	1.41 (1.29–1.54)	***< 0.001***
**Infertility Treatment Use**	*No Treatment*	1.0 (ref)	*–*	1.0 (ref)	*–*
	*Fertility Enhancing Drugs*	0.92 (0.72–1.18)	*0.562*	0.96 (0.75–1.24)	*0.800*
	*Asst. Reproductive Technology*	1.27 (1.08–1.49)	***0.003***	1.40 (1.18–1.66)	***< 0.001***
	*Both*	1.35 (0.89–2.03)	*0.146*	1.39 (0.90–2.17)	*0.134*

^a^Adjusted for sex, payment source for delivery, W.I.C, education, age, BMI, race/ethnicity, smoking, diabetes, hypertension, previous preterm birth, and infertility treatment, ^b^Reference value, ^c^non-Hispanic, ^d^Extremely obese

## Discussion

In this population-based retrospective study, based on the CDC’s annual birth data, the trend of CL/P prevalence showed a minuscule increase from 2016 to 2021. CL/P was more prevalent among mothers who were younger, NH White, AIAN, NHOPI, smoking cigarettes, and those who had pre-pregnancy diabetes, pre-pregnancy hypertension, obesity, and used infertility enhancing treatments. Also, it should be noted that the prevalence of CL/P was significantly higher among males with a male to female ratio of 1.58 to 1.

Our finding regarding the overall prevalence of CL/P from 2016 to 2021 is in accordance with the reported prevalence of CL/P by CDC in 2020 [21, 22]. This reported prevalence by CDC in 2020 was 4.95 per 10,000 live births which was slightly higher than our reported prevalence. This inconsistency is mainly due to the inclusion of the non-isolated CL/P cases as well as isolated cases in the reported prevalence by CDC. Besides CDC, the most recent study by Mai et al., examined the prevalence of major congenital birth defect from 2010 to 2014 using data from the National Birth Defects Prevention Network (NBDPN) [16]. They reported an almost two-fold higher prevalence of 10.25 for cleft lip with or without cleft palate. This difference may be mainly due to the inclusion of still births as well as live births along with non-isolated cases in the total prevalence. The prevalence of congenital anomalies is expected to be higher among still births, thus causing higher estimation of the prevalence. It should also be noted that Mai et al. have used estimative method for the calculation of the CL/P prevalence, whereas in our study, we already had the complete data for annual live births.

We examined possible risk factors associated with the occurrence of CL/P. We found that smoking before pregnancy, pre-pregnancy diabetes, pre-pregnancy hypertension, obesity, previous preterm birth, and use of assisted reproductive technology can significantly increase the risk of CL/P among American mothers. This is in accordance with the findings of previous studies. It has been shown that CL/P is one of the most frequent congenital malformations among mothers with diabetes [23–25]. One study that examined orofacial clefts among American mothers have found similar results, and reported that pregestational diabetes was significantly associated with CL/P, even after adjustment [26]. The same is the case for pre-pregnancy hypertension and previous preterm birth [27–30]. Based on a large cross-sectional study using WHO’s multicounty survey on newborn health, chronic hypertension was associated with increased risk of developing several congenital malformations including CL/P [31].

The association between pre-pregnancy smoking and increased risk for developing CL/P have been reported by several previous studies [32–36], however, our study is among the few to estimate the risk based on the frequency of maternal smoking (i.e., number of cigarettes per day) [37]. It should be noted that the odds ratios increase as the frequency of smoking increases, however, the risk suddenly diminished for mothers who smoke more than 20 cigarettes per day compared to mothers who smoke 11 to 20 cigarettes per day. Based on the results on Table 2, we observe that the difference between the odds ratios in these two groups is lower in the adjusted multivariable model compared to the univariate model. This can pinpoint the fact that this difference may alter if other confounding factors such as dietary habits, nutritional status, and alcohol consumption were available in the CDC dataset and included in our model. Because most of the high-risk behavioral habits (smoking, alcohol consumption, and dietary habits) are associated with each other and could pose a synergistic effect.

As it is evident from our results, the prevalence of CL/P-affected pregnancies was higher among younger mother and younger maternal age was associated with increased risk for developing CL/P [38, 39]. This result is in contradiction with previous studies that reported increasing maternal and paternal age are associated with increased risk of CL/P. This finding most probably points to certain missing confounding factors in the CDC data and different missing records across age groups. As it is evident from Table 2, the ORs have changed towards 1 from the first model to the second adjusted model. Perhaps certain unavailable confounding factors may have altered this result. But more importantly, the rate of missing records for CL/P among mothers older than 29 were higher compared to those who were younger than 25. This can also affect the prevalence rates in these groups. Of the 17,872 missing cases, 28.76% were 30 to 34 years old, 17.35% were 35 to 39 years old, and 16.37% were 20 to 24 years old.

Orofacial clefts are recognized to be common malformations associated with assisted reproductive technology [40–45], similar to the findings of our study. Interestingly, the odds ratio for assisted reproductive technology was the only odds ratio that increased after adjustment. One study showed a significant difference between the chance of developing CL/P among mothers undergoing assisted reproductive technology based on their maternal BMI. Mothers with obesity had higher risk for developing congenital malformations compared to mothers with normal BMI [46]. This can be the underlying cause for this increase after adjustment. Using fertility enhancing drugs was not associated with increased risk of developing CL/P in adjusted and unadjusted models.

### Strengths and limitations

To the best of our knowledge, our study is the largest national study to report the prevalence of isolated CL/P with more than 20 million live births and more than 10,000 isolated CL/P cases. More importantly, the number of missing cases was lower than 0.1% compared to previous studies on congenital birth defects. The previous study conducted by CDC data also included possible socioeconomic and metabolic confounding factors. However, our study was faced with certain limitations. CDC data did not have any information regarding dietary habits and nutritional status, alcohol consumption and history of substance abuse, familial history of CL/P or any other congenital anomalies, medication use before and during pregnancy, and type of CL/P. This could have adversely affected our statistical models.

## Conclusions

In this retrospective national study, the prevalence of isolated CL/P was 4.88 per 10,000 livebirths from 2016 to 2021. We found no significant decreasing or increasing pattern from 2016 to 2021 and the prevalence was approximately the same, albeit its slight increase in 2018. Among the prevalence was higher among mothers who were younger than 29 or older than 40 years old. The prevalence was higher among non-Hispanic White, AIAN, and NHOPI mothers. We found a significant association between pre-pregnancy obesity, pre-pregnancy diabetes, pre-pregnancy hypertension, previous pre-term birth, and use of assisted reproductive technology with increased risk of developing of CL/P. As our dataset only included livebirth, termination due to fetal anomalies are not included. Hence, the calculated prevalence may have been affected by underestimation.

### Supplementary Information

**Additional file 1: Appendix 1. **Age Pattern of CLP−affected Pregnancies from 2016 to 2021.

**Additional file 2: Appendix 2. **Race/ehtnicity Differences among CLP−affected Pregnancies from 2016 to 2021.

**Additional file 3: Appendix 3. **Smoking Pattern of CLP−affected Pregnancies from 2016 to 2021.

**Additional file 4: Appendix 4. **Use of Infertiltiy Treatment among CLP−affected Pregnancies from 2016 to 2021.

## Data Availability

The CDC dataset is publicly available at https://www.cdc.gov/nchs/data_access/vitalstatsonline.htm#Births.
