# Supplementary material for: Prevalence, trend, and associated risk factors for cleft lip with/without cleft palate: a national study on live births from 2016 to 2021
Source: BMC Oral Health. 2024 Jan 7;24:36. doi: 10.1186/s12903-023-03797-z (PMC10771673; doi:10.1186/s12903-023-03797-z)
Supplement: Supplementary file 2 — Additional file 2: Appendix 2. Race/ehtnicity Differences among CLP−affected Pregnancies from 2016 to 2021. [file 12903_2023_3797_MOESM2_ESM.pdf]

# Appendix 2. Race/ehtnicity Differences among CLP−affected Pregnancies from 2016 to 2021

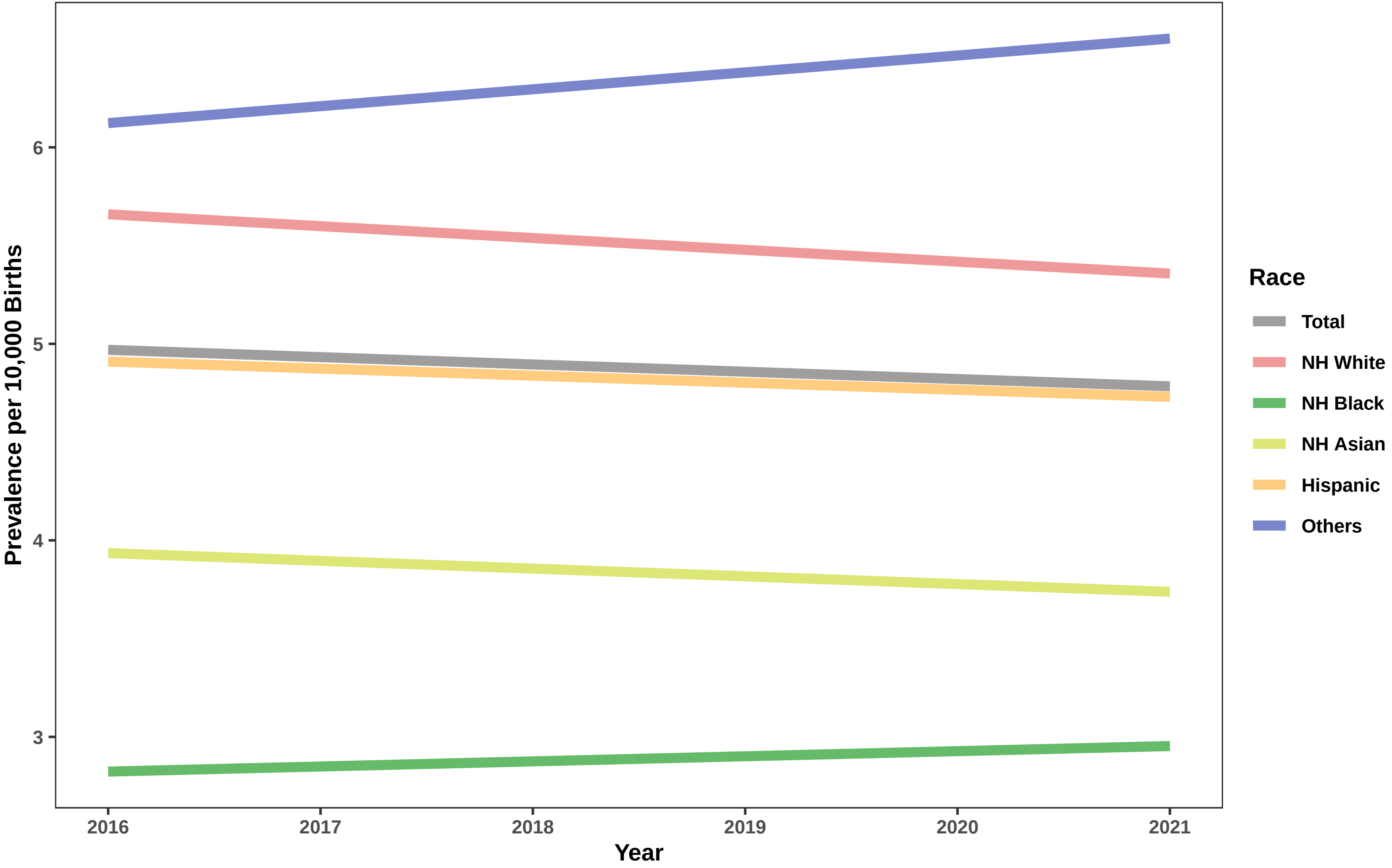


*(1) Insignificant Trend from 2016 to 2021  in All Groups ($p < 0.05$).*
*(2) Significant Difference Between All Groups ($p < 0.05$).*
*(3) NH: Non−Hispanic*
