# Supplementary material for: Prevalence, trend, and associated risk factors for cleft lip with/without cleft palate: a national study on live births from 2016 to 2021
Source: BMC Oral Health. 2024 Jan 7;24:36. doi: 10.1186/s12903-023-03797-z (PMC10771673; doi:10.1186/s12903-023-03797-z)
Supplement: Supplementary file 4 — Additional file 4: Appendix 4. Use of Infertiltiy Treatment among CLP−affected Pregnancies from 2016 to 2021. [file 12903_2023_3797_MOESM4_ESM.pdf]

Appendix 4. Use of Infertiltiy Treatment among CLP−affected Pregnancies from 2016 to 2021

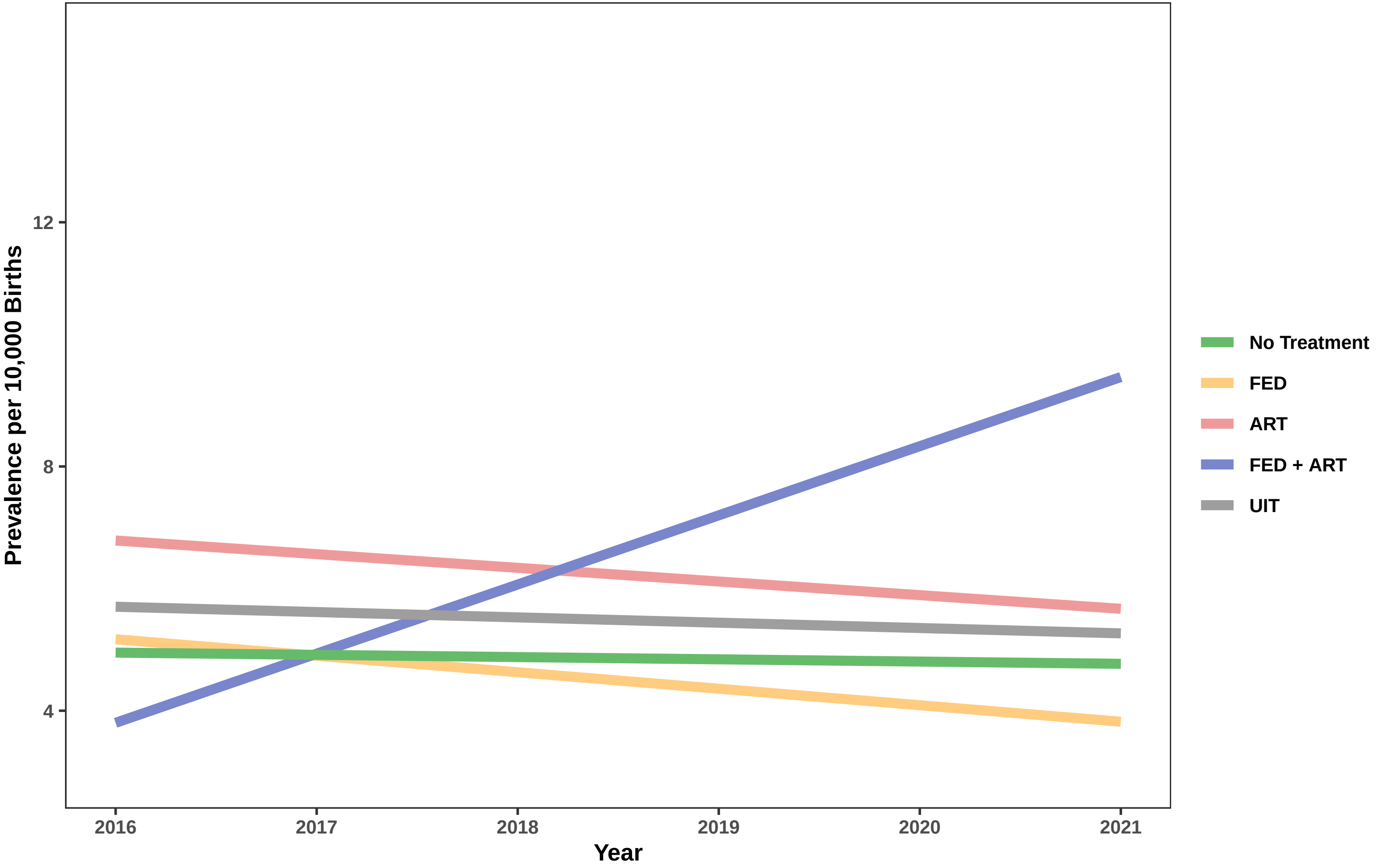

(1) Insignificant Trend from 2016 to 2021 among All Groups except Those Who Recieved No Treamtent ($p < 0.05$).
(2) Significant Difference Between All Groups ($p < 0.05$).
(3) FED: Fertlity Enhancing Drugs; ART: Asst. Reproductive Technology; UIT: Use of Infertility Treatment
